# Systemic thrombolysis for a mechanical atrioventricular valve thrombosis in a child with a single-ventricle palliation at Fontan stage

**DOI:** 10.1016/j.xjtc.2024.07.017

**Published:** 2024-07-29

**Authors:** Vladimir L. Cousin, Raphael Joye, Alice Bordessoule, Tomasz Nalecz, Veneranda Mattiello, Helia Robert-Ebadi, Pierre Fontana, Tornike Sologashvili, Maurice Beghetti, Julie Wacker

**Affiliations:** a Pediatric Cardiology Unit, Department of Pediatrics, Gynecology and Obstetrics, Geneva University Hospital, University of Geneva, Geneva, Switzerland; b Paediatric and Neonatal Intensive Care Unit, Department of Pediatrics, Gynecology and Obstetrics, Geneva University Hospital, University of Geneva, Geneva, Switzerland; c Pediatric Cardiac Surgery Unit, Surgery Department, Geneva University Hospital, University of Geneva, Geneva, Switzerland; d Pediatric Hematology and Oncology Unit, Department of Pediatrics, Gynecology and Obstetrics, Geneva University Hospital, University of Geneva, Geneva, Switzerland; e Division of Angiology and Haemostasis, Geneva University Hospitals, University of Geneva, Geneva, Switzerland

**Figure:**
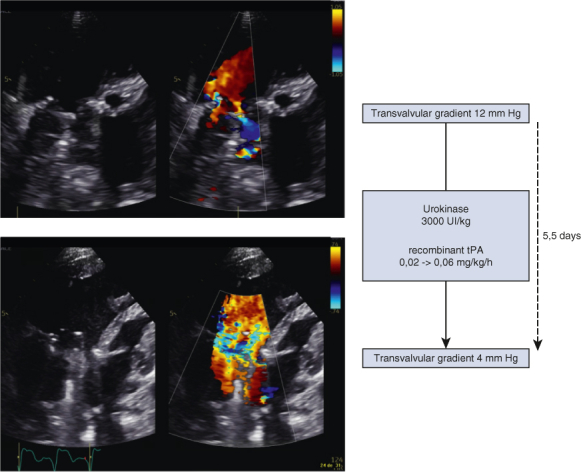
Evolution of the transvalvular gradient before and after systemic thrombolysis.

**Central Message**

Mechanical valves in univentricular congenital heart diseases are at risk of thrombosis, and thrombolysis treatment may restore valve function, avoiding redo surgery.

Good atrioventricular (AV) valve function is paramount for an effective Fontan hemodynamic and satisfactory long-term palliation outcome.^1^ Rarely, a mechanical valve needs to be inserted, carrying risk of complications, especially thrombotic events.^2^ Management options for mechanical valve thrombosis include anticoagulation, surgery, or systemic thrombolysis, as proposed in adult guidelines.^3^

In the case presentation, we illustrate the successful lysis of a mechanical valve severe thrombosis in a child under argatroban therapy due to a heparin-induced thrombopenia (HIT) early after Fontan completion.

The Institutional Review Board or equivalent ethics committee of the Geneva University Hospital approved this study by default, according to Swiss legislation regarding study of less than 5 subjects. The subject provided informed written consent for the publication of the study data.

## Case

A 3-year-old boy with hypoplastic left heart syndrome and a history of Norwood, partial cavopulmonary anastomosis, enlargement of the ascending aorta, left pulmonary artery enlargement, and previous tricuspid valve plasty and annuloplasty for severe valvular regurgitation was referred to our center for completion of total cavopulmonary anastomosis. Pre-Fontan catheterization showed a mean pulmonary artery pressure of 13 mm Hg, a mean right atrial pressure of 10 mm Hg, and pulmonary vascular resistance of 1 WUm^2^. He underwent a fenestrated Fontan operation with insertion of an ATS Medtronic 27-mm mechanical valve in tricuspid position.

**Figure 1:**
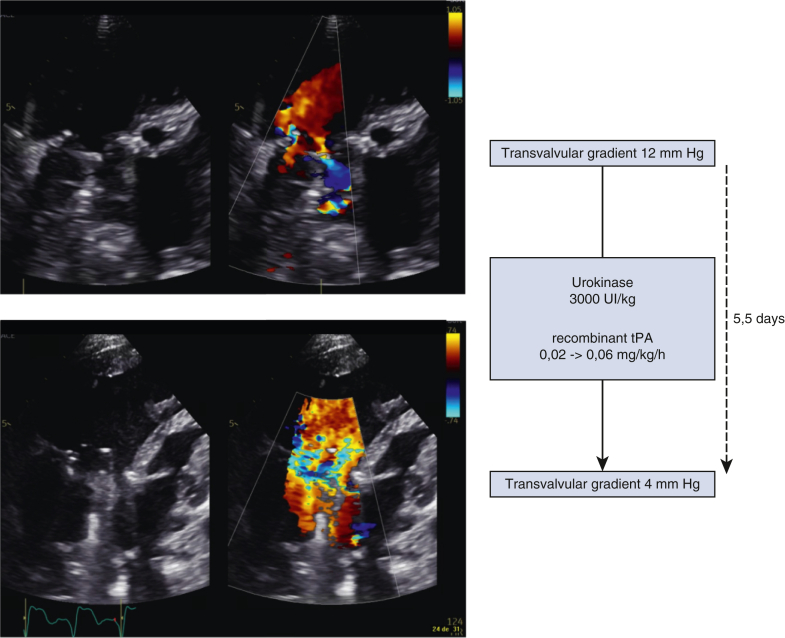
Evolution of the transvalvular gradient before and after systemic thrombolysis. *tPA*, Tissue plasminogen activator.

The patient's postoperative course was marked by altered Fontan hemodynamics with chylothorax, requiring a low-fat diet with medium chain triglycerides, and liver dysfunction (factor V activity 21% and conjugated bilirubin 21.5 μmol/L). On postoperative day (POD) 10, he developed a HIT with positive anti-PF4 antibodies (optic density 0.541), confirmed by a heparin-induced platelet aggregation test prompting a switch of anticoagulation to argatroban (Table 1). Between PODs 13 and 24, acenocoumarol was introduced progressively (target international normalized ratio [INR], 2.5-3.5), complicated by several supra-therapeutic INR levels in the context of liver dysfunction leading to transient cessation of argatroban. On POD 24, he presented a sustained desaturation with large left pleural effusion requiring pleural drain insertion and administration of a vitamin K dose for INR correction. An echocardiography showing a thrombus extending in the atrium and impairing severely the mechanical prothesis disks motion, confirmed under fluoroscopy (Figure 1), with severe AV valve stenosis (mean diastolic gradient of 12 mm Hg).

**Table 1:** Evolution of anticoagulation regimen with their target

Color of boxes with different anticoagulation Blue for Unfractioned heparin; Red for Argatroban; Grey for Thrombolysis; Green for acenocoumarol. *POD*, Postoperative day; *INR*, international normalized ratio; *rtPA*, recombinant tissue plasminogen activator.

Surgery was considered an unsuitable and high-risk option because heparin bypass would be impossible and hemorrhagic complications would be intractable with anti-IIa bypass protocol. An in situ thrombolysis with a catheter through the fenestration was contemplated but was at risk of jeopardizing the fenestration flow, and the patient had bilateral common femoral and right internal jugular vein thrombosis, making the approach of the fenestration technically challenging. After multidisciplinary discussion, systemic thrombolysis was initiated using urokinase (3000 UI/kg over 10 minutes) switched to recombinant tissue plasminogen activator (rtPA) for urokinase shortage at a dose of 0.02 mg/kg/h, progressively increased to 0.06 mg/kg/h over 24 hours. Argatroban was stopped for the first 24 hours of thrombolysis and then restarted at the same dose.

Systemic thrombolysis was continued for 5.5 days in association with therapeutic anticoagulation. Evolution was favorable (Video 1) with a progressive reduction of transvalvular gradient and restoration of mobility of the 2 disks, without any hemorrhagic complication or blood products requirement. Anticoagulation was switched to acenocoumarol, and the patient was discharged in good conditions from the hospital on POD 43. At 2 months postoperatively (POD 60), the patient was doing well, and his mean transvalvular gradient was 2 mm Hg (Figure 1).

## Discussion

We present a case of mechanical AV valve thrombosis in the context of a hypoplastic left heart syndrome post-Fontan palliation managed with medical therapy, with restoration of AV valve function after 5.5 days of rtPA therapy.

It is important to underline that the patient presented numerous risk factors for a thrombotic event: a mechanical AV valve in a Fontan physiology, a prothrombotic setting with HIT, liver dysfunction, chylothorax, and variations in anticoagulation regimen.

AV valve dysfunction is a known factor for morbidity and mortality in patients with single-ventricular physiology.^1^ Use of mechanical valve in this context remains rare, usually undertaken as a last option: A Canadian group reported 16 patients over a period of 24 years, and a Japanese nationwide study reported 56 patients over 6 years.^2^^,^^4^ Of note, 16% to 31% of the patients experienced valve thrombotic complication.^2^^,^^4^

Mechanical valve thrombosis occurs with varying incidence in the literature, and data are lacking in pediatric patients.^3^ Management options for mechanical valve thrombosis include systemic anticoagulation, surgery, or systemic thrombolysis.^3^ Different risks exist between those options and should be weighted, including the impact of a new bypass surgery or in the case of lysis, the risk of bleeding and embolism from the valve clot fragments. In our case, surgery was deemed too hazardous, in terms of bleeding risk after 6 sternotomies, and with a cardiopulmonary bypass anticoagulation with anti-IIa (bivalirudin or argatroban) without any reliable pediatric protocol. Based on adult trials results, low-dose rtPA using a prolonged infusion seems to be as effective as high-dose rtPA or surgery with less complications.^3^ Following those results, we used a similar prolonged infusion of low-dose rtPA with close monitoring of fibrinogen and platelets, as described for the pediatric population.^5^ Therapeutic anticoagulation was also promptly reinitiated after 24 hours. A close monitoring of AV valve function allows to control the therapeutical efficacy and eventually lead to dose escalation in the absence of any improvement or early cessation in case of excellent result. Interestingly, the patient presented no major or minor hemorrhagic complication despite recent surgery and the presence of multiple indwelling catheters.

## Conclusions

Mechanical AV valve placement in univentricular heart physiology carries a high risk of thrombotic complications and should remain a last option. Thrombolysis treatment in such cases should be discussed on a case-by-case basis because it may restore the valve function rapidly and without surgery.

## Conflict of Interest Statement

The authors reported no conflicts of interest.

The *Journal* policy requires editors and reviewers to disclose conflicts of interest and to decline handling or reviewing manuscripts for which they may have a conflict of interest. The editors and reviewers of this article have no conflicts of interest.

## Supplementary Data

Video 1 Capture of transthoracic echography showing the evolution of the mechanical valve at thrombus diagnosis and the evolution after treatment. Video available at: https://www.jtcvs.org/article/S2666-2507(24)00327-4/fulltext.
