# Supplementary figures and images for: Systemic thrombolysis for a mechanical atrioventricular valve thrombosis in a child with a single-ventricle palliation at Fontan stage
Source: JTCVS Tech. 2024 Jul 29;27:142–5. doi: 10.1016/j.xjtc.2024.07.017 (PMC11518958; doi:10.1016/j.xjtc.2024.07.017)

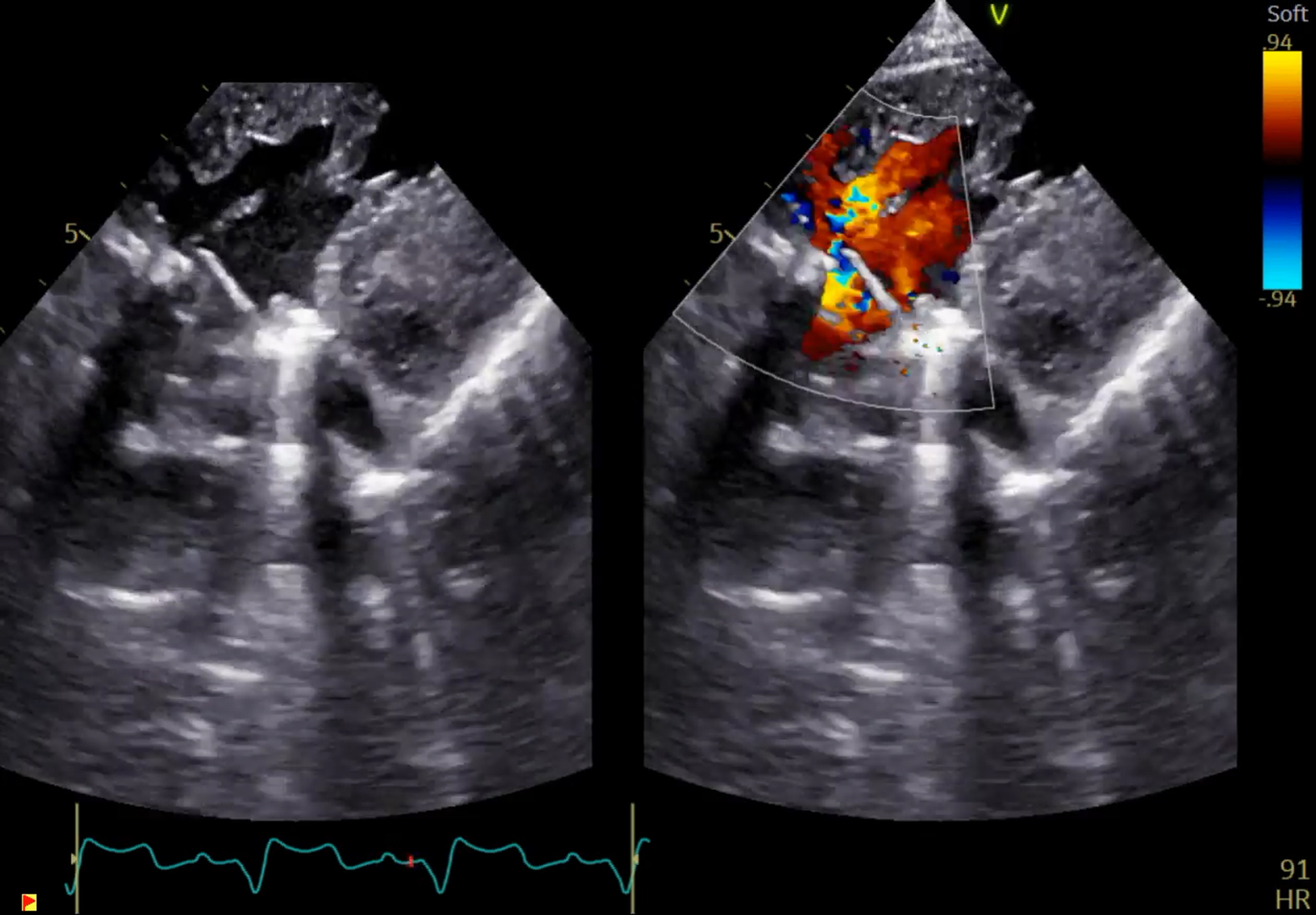

Supplement: Video 1 — Capture of transthoracic echography showing the evolution of the mechanical valve at thrombus diagnosis and the evolution after treatment. Video available at: https://www.jtcvs.org/article/S2666-2507(24)00327-4/fulltext. [file fx2.jpg]
